# New Models to Predict the Acute and Chronic Toxicities of Representative Species of the Main Trophic Levels of Aquatic Environments

**DOI:** 10.3390/molecules26226983

**Published:** 2021-11-19

**Authors:** Cosimo Toma, Claudia I. Cappelli, Alberto Manganaro, Anna Lombardo, Jürgen Arning, Emilio Benfenati

**Affiliations:** 1 Laboratory of Environmental Chemistry and Toxicology, Department of Environmental Health Science, Istituto di Ricerche Farmacologiche Mario Negri IRCCS, Via Mario Negri 2, 20156 Milano, Italy; cosimotoma88@gmail.com (C.T.); ci.cappelli@gmail.com (C.I.C.); emilio.benfenati@marionegri.it (E.B.); 2 Kode Chemoinformatics s.r.l.-Via Nino Pisano 14, 56122 Pisa, Italy; a.manganaro@kode-solutions.net; 3 Umweltbundesamt-German Federal Environment Agency, Wörlitzer Platz 1, 06844 Dessau-Roßlau, Germany; juergen.arning@uba.de

**Keywords:** quantitative structure-activity relationship (QSAR), applicability domain, *Raphidocelis subcapitata*, *Daphnia magna*, fish, biological databases, random forest

## Abstract

To assess the impact of chemicals on an aquatic environment, toxicological data for three trophic levels are needed to address the chronic and acute toxicities. The use of non-testing methods, such as predictive computational models, was proposed to avoid or reduce the need for animal models and speed up the process when there are many substances to be tested. We developed predictive models for *Raphidocelis subcapitata*, *Daphnia magna*, and fish for acute and chronic toxicities. The random forest machine learning approach gave the best results. The models gave good statistical quality for all endpoints. These models are freely available for use as individual models in the VEGA platform and for prioritization in JANUS software.

## 1. Introduction

About 300 million tonnes of chemicals that are used in consumer and industrial products are discharged into wastewaters and find their way into natural waters every year. Additional pollution comes from diffuse sources in agriculture, where it is estimated that about 140 million tonnes of fertilizers and several million tonnes of pesticides are applied annually [1]. Therefore, aquatic communities are exposed to many chemicals that can be toxic for them and humans [2,3], even in low concentrations. The management of pollution from the release of synthetic chemicals has become a cause for concern for the scientific community, regulators, and the public [4].

Risk assessment of chemicals is necessary to prevent and control pollution due to anthropogenic chemicals [5]. Information on aquatic toxicity is needed to assess the hazards and risks of chemicals to freshwater organisms. Ideally, all aquatic organisms should be tested, and the most sensitive species should be selected for assessment to protect all aquatic organisms. However, since it is impossible to test the toxicity of chemicals on all aquatic organisms one by one, only representative aquatic organisms are selected. Algae, crustaceans, and fish belong to different trophic levels, i.e., primary producers and primary and secondary consumers, and are considered relevant for the protection of aquatic ecosystems [6].

Besides hazard and risk assessments, toxicity data from these representative organisms are used for the prioritization of chemicals, which are accepted by regulatory authorities, for instance, to screen persistent, bioaccumulative, and toxic chemicals [4].

Within regulatory contexts, standard test methods for representative species that were established decades ago and continuously updated are required. Based on these protocols, the effect of a chemical can be expressed for short- and/or long-term exposure.

According to the Registration, Evaluation, Authorization, and Restriction of Chemicals (REACH) Regulation [5], ecotoxicological information is required for substances in relation to the annual amounts that are manufactured or imported in the EU. This includes short- and long-term toxicity testing on crustaceans (the preferred species is *Daphnia magna*), algae, and fish species [6]. Ecotoxicological information on these three trophic levels serves to identify the concentration of the substance below which no effects are expected in the environmental sphere of concern. This is the predicted no effect concentration (PNEC). Based on the available information, the PNEC for each environmental compartment is established. The PNEC can be calculated by applying an appropriate assessment factor to the short- and long-term toxicity data. An assessment factor is applied because only a limited number of representative species is used to extrapolate the PNEC for aquatic ecosystems.

In general, the more data there are and the longer the duration of the tests, the smaller is the degree of uncertainty and the assessment factor. An assessment factor of 1000 is typically applied to the lowest of three short-term toxicity values for species representing the different trophic levels, and a factor of 10 to the lowest of three long-term toxicity values for species representing these levels. The PNEC is then used for risk characterization [5].

The regulation for classification, labeling, and packaging (CLP) [7] recognizes that the intrinsic hazard to aquatic organisms is due to the superposition of acute and chronic hazards of a substance. The classification system uses the lowest available toxicity values between and within the different trophic levels (algae, crustaceans, and fish) to establish the suitable hazard category. The classification system for aquatic toxicity has one acute hazard classification category and three separate chronic hazard classification categories. If there is no chronic aquatic toxicity data, acute aquatic toxicity data are combined with environmental fate data, such as degradability and bioconcentration factors, to assess the chronic hazard of a substance. To ensure a protective hazard assessment, there is also a ‘safety net’ category for substances that have no data that are useful for classification in the other categories, but there are nevertheless some grounds for concern [7].

Annex XIII of the REACH Regulations [5] sets criteria for substances that are persistent, bioaccumulative, and toxic (PBT) or very persistent and very bioaccumulative (vPvB). The PBT/vPvB assessment is required for all substances that are manufactured or imported in amounts of 10 tonnes or more per year. The PBT assessment is one of the criteria that are used in REACH for the prioritization of chemicals [5]. Substances that persist for long periods in the environment and have a high potential to accumulate in biota are of specific concern because exposure to these substances is very hard to predict. Their persistence means that these substances may not degrade near emission sources, and they may be gradually transported into remote areas (long-range transport) and may exert their toxic effect for a long time, even if emissions are stopped [8]. The chronic toxicity of chemicals toward aquatic organisms is used to assess the fulfillment of the toxicity criterion. In the phase of screening for PBT, the short-term toxicity toward aquatic organisms from the three trophic levels is also considered. Thus, in many regulatory contexts, both the acute short-term and long-term toxicity data of aquatic organisms are necessary.

Even though there are established protocols for aquatic toxicity tests, acute short-term and chronic aquatic toxicity data for many chemicals are still scarce [9] because generating in vivo data is time-consuming and expensive. However, the REACH Regulation encourages the use of quantitative structure-activity relationships (QSARs) to provide information about the hazards that are associated with chemicals, also within a weight of evidence approach, since computational methods are considered a rapid and low-cost option and might be also useful to reduce the number of chemicals to be tested in vivo [10].

Many local and global QSAR models were developed to predict the toxicity of chemicals toward algae, the crustacean *Daphnia magna*, and fish species. The local models performed well for a limited chemical domain but were not useful to assess a diverse set of chemical structures [11]. Most of the QSAR models focus on acute aquatic toxicity [12,13,14,15,16,17,18,19,20,21] and only a few relate to chronic aquatic toxicity. Claeys et al. [22] and Austin and Eadsforth [23] developed models for chronic narcosis toxicity and chronic non-polar narcosis toxicity in fish, respectively. Fan et al. [24] developed a local model that was based on 48 substituted benzenes to predict the chronic toxicity of chemicals toward *Daphnia magna*. Ding and colleagues [4] developed classification models to predict the chronic toxicity of chemicals toward *Daphnia magna* and the green algae *Pseudokirchneriella subcapitata*. ECOSAR software contains chronic toxicity models for algae, daphnids, and fish [25]. Several QSAR models, such as those included in ECOSAR, were used to predict the toxicity of chemicals, but they yielded poor correlations and still need improvement [6]. QSARCHE contains non-polar and polar narcosis QSAR models for chronic toxicity in fish [26]. However, given the current state of development of chronic aquatic toxicity models, considerably more work is still needed [27].

The objective of the present work was to develop QSAR models to predict the acute and chronic toxicity of various chemicals toward algae (*Raphidocelis subcapitata*, previously known as *Pseudokirchneriella subcapitata*), *Daphnia magna*, and fish (several species) based on OECD Good Laboratory Practice (GLP) data [28]. It is expected that these models will help to fill gaps in the information on the acute and chronic toxicity of several chemicals for all the trophic levels of freshwater ecosystems.

This work is a part of a project called JANUS (Joining Environmental, Ecotoxicological, and Toxicological Assessment of Chemical Substances with Non-Testing Methods within a Unified Screening), funded by the Bundesministerium für Umwelt, Naturschutz und nukleare Sicherheit (BMU), that aims for the implementation of a new strategy for the prioritization of chemicals according to PBT; endocrine disruption; and carcinogenic, mutagenic, and toxic-to-reproduction (CMR) properties.

The QSAR models described here are used to assess the ecotoxicity of chemicals within the toxicity criterion of the JANUS prioritization scheme. They advance the work done in a previous project, called PROMETHEUS [29], which was based on calculations of the acute-to-chronic ratio (ACR) to check the fulfillment of the toxicity criterion for PBT assessment.

## 2. Results

We checked a large number of substances with experimental values on organisms representing the three trophic levels, as required by the European regulation [5], for acute and chronic values. This collection of values is large compared with other collections, particularly on chronic toxicity. Chronic toxicity is the most important value according to the European regulation [5], and the acute value has to be calculated only if information regarding the chronic value is missing. We applied a quality check regarding the chemical structures and the consistency of property values when more than one value was found. The presence of multiple values offers an advantage, increasing the reliability of the experimental value. However, when there are significant differences between the experimental values, this increases the uncertainty and may indicate the presence of errors or difficulties with a certain substance. For this reason, we adopted the procedure described to prune the dataset, excluding substances with uncertain experimental values.

Then, we developed QSAR random forest models (the tree ensemble method) for three trophic levels—*Raphidocelis subcapitata*, *Daphnia magna,* and fish—for both acute and chronic toxicities. We made the distribution of the endpoint data uniform and split the dataset to get a training and a validation set. We calculated the descriptors and pruned them using a genetic algorithm (gaselect) or variable selection using random forest (VSURF) approaches. For each model, we checked different parameters to identify ideal thresholds for the applicability domain (AD) and examined them in every possible combination. We assessed the performance by including and excluding high leverage compounds. A total of thirty-two possible AD combinations were evaluated; then, the best one was selected based on the best compromise in terms of coverage and performance in 10-fold cross-validation. Each model was validated on an external set of data.

Table 1 summarizes the statistics for the training and the external validation sets, with the variable selection method chosen for each model, the number of descriptors, and details of the parameters used to define the AD. For each endpoint and trophic level, the table also shows the statistics with and without the AD. E(L)C_50_ represents the acute toxicity, i.e., the concentration necessary to produce the effect (death) in half of the exposed population, whereas the no observed effect concentration (NOEC) represents the chronic effect.

The statistics were evaluated using the determination coefficient (R^2^), the mean absolute error (MAE), and the root-mean-squared error (RMSE). All models gave high statistics for the training set. This was expected since the random forest approach was adopted. The most interesting values were those related to the validation set, which contained substances that were never used to build up the model (about 20% of the total number of substances available). These values provided a good assessment of the expected predictivity of the models.

The model for *Raphidocelis subcapitata* acute toxicity (EC_50_) gave an R^2^ of 0.60 for the validation set. Though this does indicate uncertainty in the prediction, it is still acceptable for this kind of endpoint. The use of the information on AD did not substantially improve the predictions. The models for *Raphidocelis subcapitata* gave the worst results. However, the results were worse toward acute toxicity.

The model for *Daphnia magna* acute toxicity (EC_50_) gave an R^2^ of 0.69 for the validation set. Although this indicates uncertainty in the prediction, it is still acceptable for this kind of endpoint. Information on the AD did not substantially improve the predictions. In the case of models for NOEC, the R^2^ in the validation sets was even better at 0.78, and this is useful, also because this endpoint is much less studied than acute toxicity.

The results for fish were comparable to those for *Daphnia magna*, with a small reduction in the statistical quality. Furthermore, in fish, there was an improvement in performance regarding chronic toxicity. This is a good result too.

## 3. Discussion

We developed a battery of models for three trophic levels that are important for aquatic toxicity: *Raphidocelis subcapitata*, *Daphnia magna*, and fish for both acute and chronic toxicities. The statistical parameters were good, and similar or better than those that were previously published.

For the algal acute toxicity, too few models are available, and the majority are for specific classes of chemicals [30,31]. This can explain the better statistics compared to ours.

The models for chronic toxicity toward *Raphidocelis subcapitata* and algae in general are very limited. Ding et al. [4] developed classification models, which are quite different from the regression models that predict continuous values. However, they obtained worse models than the classification models toward *Daphnia magna*, and this confirmed the greater difficulty in predicting this endpoint.

Considering the low number of available models for algal toxicity (acute and chronic), it is clear that more efforts are needed to develop new models and have a sufficient number of models to better assess these endpoints. We explained how using more than one model for the same endpoint may help the user in the assessment [32].

While many models were published for fish and *Daphnia magna* acute toxicities, the number of studies for chronic effects is much scarcer.

Recent studies on *Daphnia magna* acute toxicity had similar difficulties to those reported above and achieved R^2^ values of about 0.60–0.68 for the validation set [33,34]. However, Khan and colleagues used 175 [33] and 133 [34] compounds; therefore, our model used a much larger set of compounds: 428. Thus, it is quite probable that the present model is more robust and has a larger AD than the previous ones. We also noticed that the model by Khan et al. [34] is specific for biocides, and this might explain the lower R^2^, and the other model [33] is for pharmaceuticals. If we consider older models, their performance was less satisfactory [35].

Few models about the chronic toxicity for *Daphnia magna* have been published. The model developed by Fan et al. [24] is a multilinear regression model with five descriptors and gave an R^2^ for the test set of 0.736. However, this model is quite different from the one we developed because this is only for substituted benzenes and is validated on ten substances.

Many models have been published for acute fish toxicity. Older models gave worse performances [36]. More recent models achieved similar or even better results. For instance, the models by Khan et al. [33] achieved an R^2^ of 0.8 for the validation set.

Very few models have been published about fish chronic toxicity. Claeys et al. [22] developed a model for nonpolar narcosis based on 49 substances for the training set and 20 for the test set. LogP was the key parameter. Even fewer substances were available for polar narcosis (13). The R^2^ for the training and test sets for nonpolar narcosis were 0.76 and 0.73, respectively, which were quite similar to the R^2^ of 0.74 of the ECOSAR model for neutral organics [37]. However, these values refer only to nonpolar substances without a specific mode of action or reactivity. As we said, the model for polar narcosis had only a very small number of compounds, and the user needs a previous tool to see whether the substance of interest follows the narcosis mechanism; for the other cases, the substances were outside the AD of the system. Both the ECOSAR and the QSARCHE (the software developed by Claeys et al. [22]) were criticized later by Austin and Eadsforth [23] for the use of some inaccurate data in these models. They proposed another model for NOEC for nonpolar narcotics, which achieved an R^2^ of 0.89 for the test set [23]. There were 10 substances in the test set and 19 in the training set. Thus, for this model too, there are the general limitations of the small number of substances at the basis of the model and the need to know or predict whether the target substance is a nonpolar narcotic.

In general, there is the need for further studies addressing aquatic toxicity for the three trophic levels for both acute and chronic toxicities. This study represents a contribution in this direction.

The models we describe can be applied for the assessment of chemical substances in a facilitated way because they were implemented within the VEGAHUB tools [38]. One is VEGA, which is a freely available platform that contains dozens of models. The availability within the same system of the QSAR models, of the software assessing the applicability domain for each substance, and a tool to visualize the six most similar substances for read-across offers great improvements compared to the past situation. This provides a way to evaluate the confidence of the prediction for the specific chemical. The other tool is JANUS, which is freely available software that was designed to assess and prioritize the chemicals according to their PBT properties.

## 4. Materials and Methods

### 4.1. The Datasets

We collected the experimental toxicity values from short- and long-term aquatic toxicity tests of chemicals that were conducted by the Japanese Ministry of Environment on several organisms; these have been publicly available on the official website [39] since March 2016.

The aquatic toxicity tests were done according to the OECD GLP standards [28] and the OECD official guidelines for several species and endpoints [40,41,42,43,44]. In view of the purposes and the context of the JANUS project, we selected the following representative organisms of the three trophic levels of freshwater ecosystems:

- Algae (*Raphidocelis subcapitata*, previously known as *Pseudokirchneriella subcapitata*): EC_50_ 72 h (growth rate);
- Algae (*Raphidocelis subcapitata*, previously known as *Pseudokirchneriella subcapitata*): NOEC 72 h (growth rate);
- Daphnids (*Daphnia magna*): EC_50_ 48 h, acute effect (immobilization);
- Daphnids (*Daphnia magna*): NOEC 21 d, chronic effect (reproduction);
- Fish (*Oryzias latipes*): LC_50_ 96 h, acute effect (mortality);
- Fish (*Oryzias latipes*): NOEC, chronic effect, as in the early-life stage toxicity test [43].

We generated the chemical structures of the compounds as SMILES notations [45] from the chemical name and CAS RN using ChemCell [46] and Marvin View 17.12, 2012017, ChemAxon [47]. We manually checked the correctness and consistency of the chemical structures, chemical names, and CAS RN using several databases, including ChemIDplus Advanced [48], PubChem [49], ChemSpider [50], and DSSTox [51]. We added the structures that were not automatically generated using these databases.

Then we pruned the initial datasets. This was done mostly because classical QSAR approaches, particularly for the calculation of molecular descriptors, cannot deal with certain types of structures (inorganic, disconnected structures like salts, etc.). We excluded metal complexes, inorganics, mixtures of structural isomers, ambiguous structures, non-ionic surfactants, mixtures, complex disconnected structures, chemicals whose names and CAS RN did not correspond, and “substances of unknown or variable composition, complex reaction products, or biological materials” (UVCBs). We also neutralized the salts.

As the next step, we selected continuous experimental values, excluding those reported as a range, as above or below a certain numerical threshold, or only approximate. We kept the toxicity values from experimental conditions of the assays as they are defined in the OECD guidelines [40,41,42,43,44]. For instance, we excluded toxicity values from 0–48 h assays on *Raphidocelis subcapitata* and LC_50_ for fish after 120 h of exposure. We also eliminated pH-adjusted toxicity values for fish and *Daphnia magna*. We calculated the molecular weight of each chemical structure to convert the experimental toxicity value from milligrams per liter to millimoles per liter.

We checked the multiple values for the same species and endpoint for each substance: the difference between the largest and the smallest values had to be within a factor of 10, i.e., one log unit, when the experimental conditions and the reliability of the studies were the same, as reported in [52]. When possible, we looked for the outlier(s); if not found, we eliminated the data and the substance was not used. We also checked whether the experimental toxicity values were higher than the water solubility. If it was so, we eliminated this chemical.

Once the dataset was pruned, we obtained the number of chemicals for the different trophic levels and endpoints that are reported in Table 2.

Since after the pruning, the Japanese Ministry of Environment dataset contained only 35 chemicals for chronic fish toxicity, we searched other sources of experimental data for this endpoint. We retrieved many experimental data from the ECOTOX Aquire database [53], which was updated in July 2017, and pruned this dataset according to several criteria:

- Taxonomic: animals, fish, and standard test species.
- Test results: endpoint (NOEC) and effect measurement (mortality).
- Test conditions: test location (laboratory), exposure media (freshwater), and exposure types (flow-through, renewal).
- Chemical analysis: measured.
- Purity > 80% and “not reported”; if the purity was “not reported,” we checked the chemical grade (eliminated: experimental, practical, and technical grades).
- Organism life stage: egg(s), embryo(s), blastula, eyed egg or stage, and eyed embryo.
- Organism age: *Pimephales promelas* by 5 d, *Danio rerio* by 5 d, and *Oncorhynchus mykiss* by 35 d.
- Number of doses: 4 or more.
- Duration: 28 d post-hatch (*Pimephales promelas*), 30 d post-hatch (*Danio rerio*), and 60 d post-hatch (*Oncorhynchus mykiss*).
- Inorganics were eliminated.

A further source of data was the PROMETHEUS dataset on chronic fish toxicity [29], which included experimental data from eChemPortal [54] and several datasets that were extracted from OECD QSAR Toolbox 3.2 [55].

We used the same criteria as above to check the data from these two additional sources. We merged all the datasets on chronic fish toxicity from various sources [39,53,54,55], checking multiple values and duplicates. The final chronic fish toxicity dataset contained 94 chemicals.

We calculated the median and the arithmetic and geometric means of the multiple values in millimoles per liter to check whether there were differences between them to integrate the multiple values. We found a very good correlation (R^2^ ≈ 1) and we chose the geometric mean, as recommended by [52]. We calculated the logarithm of the geometric mean to normalize the data. We also tried a Box–Cox transformation [56], optimizing the ʎ value for each dataset. Since the Box–Cox transformation gave better results in terms of normalization of the data, we used this instead of the logarithm to normalize the data. We excluded data that fell outside the range (mean of the Box–Cox transformed values) ± 3 SD (standard deviation).

For each dataset, we normalized the SMILES using istMolBase 1.0.3 [57]. We neutralized the normalized SMILES of the compounds in the datasets of acute and chronic toxicity endpoints for *Daphnia magna* and fish. We used ionized normalized SMILES in the case of EC_50_ 72 h and NOEC 72 h datasets for *Raphidocelis subcapitata* since pH is a critical issue in algae [41]. According to the OECD guideline [41], two growth media can be used: one at pH 7.5 and another at pH 8.1. The two growth media can give different results, depending on the pH, especially for ionizing substances [41]. We had no information on what medium was used to perform the assays; therefore, we calculated the main microspecies at pH 7.5 and 8.1 for all the SMILES using JChem [58] and eliminated compounds with SMILES that changed depending on pH. The dataset containing the CAS RN, the SMILES, and the experimental values of each model are included as Supplementary Material (FinalDataset.xls).

### 4.2. The QSAR Models

To build up the models, we divided the datasets into training and test sets in a ratio of 80:20. To obtain a uniform distribution of the endpoint values between the two subsets, we applied an activity and descriptors sampling method. First, principal component analysis (PCA) was done on all the 2D descriptors that were calculated using the Dragon 7.0 Extension for KNIME [57,59]; the first two principal components were selected. Five random compounds were selected. Then, we chose the most dissimilar compound from the sample pool according to the first two principal components and the response using several combinations of distance metrics and scoring functions. Then, this compound was added to the pool and the operation was repeated until the desired number of compounds for the training set was reached (80% of the substances).

As explained before, the Dragon 7.0 Extension for KNIME was used to calculate 2D descriptors, excluding compounds whose Ghose-Crippen octanol-water partition coefficient [60] (ALOGP) could not be calculated (because this descriptor was the one that was most closely correlated to the response). Dragon 7.0 can calculate 3839 2D descriptors. A large part of this number of descriptors is likely to be redundant or not informative; therefore, one must apply methods to reduce the variables to train the model (pruning). The procedure adopted was divided into three phases:

1. All the descriptors with constant values (var(X) = 0) were eliminated;
2. All the descriptors that correlated higher than 0.95 (Pearson) with at least one other descriptor were eliminated;
3. A genetic algorithm (gaselect) or variable selection using random forest (VSURF) was applied.

For each dataset, we had two pools of variables, one selected with gaselect and the other with VSURF. Both datasets were imported into a KNIME workflow to derive the models.

Among the several algorithms used, a random forest (RF) called tree ensemble gave the best results in terms of performance. This algorithm builds a series of regression trees with different rows and different variables (according to certain parameters) and then the results are aggregated as an ensemble of models. The parameters for the variables of each tree and the number of compounds are selected on the basis of the performance of several models (hyperparameter-tuning research) using R^2^ as a metric of the bootstrap (100 iterations) cross-validation on the training set.

Two approaches were applied to define the applicability domain (AD) of the models:

1. The first approach explored the structural domain of the model. This was done by recording the degree of structural similarity of a given compound to those in the training set. A distance matrix containing distances for each pair of compounds in the training set was calculated; then, for each compound in the training set, we calculated the mean distance to its first k neighbors. The training set chemicals were then sorted on the basis of these distances and the value corresponding to a given percentile of the distribution of distances was used as a threshold (T_D_), beyond which, chemicals were excluded from the AD. For the external validations, the procedure was repeated, calculating the distance of each validation set chemical from their neighbors in the training set; then, T_D_ was used to identify the compounds outside the AD. For the present work, we used the Euclidean and Manhattan distance metrics; values assigned to k were 1 and 5; values assigned to T_D_ were those corresponding to the 100th, 97.5th, 95th, and 90th percentiles of the training set distance distribution.

2. The second approach was based on the derivation of a so-called “error model”, which predicts the uncertainty of the predictions from an “activity model”. An activity model is a classical model that is based on chemical descriptors as independent variables and an endpoint (e.g., a biological activity) as the dependent variable. An error model is derived from the same training set as the associated activity model; the differences from the cross-validated absolute errors (previously generated by the activity model) are the dependent variables, while the independent variables are a series of AD metrics that reflect the accuracy of the predictions that are made by the activity model. Six AD metrics were used as “descriptors”: wRMS1 is the weighted root-mean-squared difference between the predicted activity of the target and the observed activity of its five neighbors in the training set; wRMS2 is the weighted root-mean-squared difference between the predicted activity of the five neighbors of the target and the observed activity of the same neighbors; SIMILARITYNEAREST1 and SIMILARITYNEAREST5 are the Euclidean distance of the target from one and five neighbors in the training set, respectively; TREE_SD is the standard deviation of the prediction of the target among the RF trees; and PREDICTED is the prediction for the target. The RF algorithm was used for the error model. Training set chemicals were sorted based on errors in the predictions that were estimated by the error model; then, the value corresponding to a given percentile of the distribution of predicted errors was used as a threshold (T_E_), beyond which, chemicals were excluded from the AD. The same T_E_ was applied to the predicted errors that were calculated for the validation set chemicals. For the present work, the values that were assigned to T_E_ were those corresponding to the 100th, 90th, 75th, and 65th percentiles of the training set error distribution.

## Figures and Tables

**Table 1:** The statistical parameters of the QSAR models on aquatic toxicity. Box-Cox transformation of millimoles per liter was used in place of the logarithm of millimoles per liter.

		*R. subcapitata*		*Daphnia magna*		Fish	
		EC_50_^a^	NOEC ^b^	EC_50_^a^	NOEC ^b^	LC_50_^a^	NOEC ^b^
Training set	R^2 c^	0.96	0.95	0.94	0.95	0.95	0.96
	MAE ^d^	0.41	0.56	0.49	0.42	0.27	0.54
	RMSE ^e^	0.52	0.74	0.64	0.56	0.37	0.68
Validation set without AD ^f^	R^2 c^	0.59	0.58	0.56	0.74	0.65	0.74
	MAE ^d^	0.96	1.36	0.99	0.83	0.68	1.75
	RMSE ^e^	1.25	1.73	1.31	1.13	0.87	2.45
Validation set with AD ^f^	R^2 c^	0.6	0.63	0.69	0.78	0.65	0.76
	MAE ^d^	0.97	1.29	0.84	0.8	0.64	1.79
	RMSE ^e^	1.26	1.66	1.09	1.07	0.83	2.54
	Coverage	0.89	0.93	0.84	0.81	0.81	0.89
Details of the model	Feature selection	VSURF	GASELECT	GASELECT	VSURF	VSURF	GASELECT
	No. of descriptors	13	40	12	17	13	12
	Distance mode	Euclidean-5	Euclidean-5	Euclidean-1	Euclidean-1	Euclidean-5	Euclidean-5
	Distance threshold	0.9	0.975	0.9	0.975	0.9	0.975
	Error percentile	0.9	1	0.75	0.75	1	1

^a^ E(L)C_50_ is the concentration that causes the effect (death) in 50% of the exposed population. ^b^ NOEC is the no observed effect concentration. ^c^ R^2^ is the determination coefficient. ^d^ MAE is the mean absolute error. ^e^ RMSE is the root mean squared error. ^f^ AD is the applicability domain of the model.

**Table 2:** Number of chemicals for each trophic level from acute short-term and chronic toxicity tests available in the Japanese Ministry of Environment’s database after pruning of chemical structures and experimental values. The numbers of substances before pruning are given in parentheses.

	Acute Toxicity Test	Chronic Toxicity Test
Number of chemicals for *Raphidocelis subcapitata*	315 (372)	408 (577)
Number of chemicals for *Daphnia magna*	428 (509)	306 (372)
Number of chemicals for fish	331 (393)	35 (37)

## Data Availability

The substances and the property values used to build up the models are freely downloadable from the VEGA system (www.vegahub.eu), for each model, where the user chooses the model to run.

## Supplementary Materials

The following are available online. Excel S1: FinalDataset.xls.

## Sample Availability

Samples of the compounds cited in this article are not available from the authors.
